# Synergistic enzyme action boosts phenolic compounds in flaxseed during germination using a two-level factorial design

**DOI:** 10.1038/s41598-025-25059-4

**Published:** 2025-11-18

**Authors:** Amal Z. Barakat, Azza M. Abdel-Aty, Hala A. Salah, Roqaya I. Bassuiny, Saleh A. Mohamed

**Affiliations:** Molecular Biology Department, National Research Centre, Dokki, Cairo Egypt

**Keywords:** Flaxseed, Germination, Phenolic compounds, Antioxidant activity, Endogenous enzymes, Statistical modeling, Biochemistry, Biotechnology

## Abstract

A two-level factorial design in Design-Expert^®^ software was applied to statistically model and optimize the effects of key endogenous enzymes on the release of total phenolic content (TPC) and total flavonoid content (TFC) during flaxseed germination. The model demonstrated high predictive accuracy (R² > 0.99) and identified germination day (GD), β-glucosidase (β-GL), peroxidase (POX), and catalase (CAT) as critical variables with significant synergistic interactions influencing TPC and TFC biosynthesis. Under these optimal factors, five-day germinated sprouts showed the highest levels of bioactive compounds, with TPC and TFC increasing by 7.6-fold and 38.27-fold, respectively. Compared to dry seeds, High-performance liquid chromatography (HPLC) analysis confirmed marked increases in sinapic acid (6.4-fold), gallic acid (6.1-fold), and p-coumaric acid (5.5-fold). Antioxidant activity also improved, as evidenced by reduced IC_50_ values for DPPH (2.26-fold) and ABTS (2.6-fold) assays. Enzyme activity analysis revealed early-stage activation of polyphenol oxidase (PPO) and phenylalanine ammonia-lyase (PAL), supporting the enzymatic role in phenolic compound biosynthesis. Additionally, 5-day sprout extracts exhibited notable antibacterial and antidiabetic activities. These findings provide a robust, model-guided framework for enhancing flaxseed’s nutritional and functional value through controlled germination, with direct applications in the development of health-promoting functional foods.

## Introduction

Flaxseed (*Linum usitatissimum*), an ancient crop from the Linaceae family, is renowned for its health benefits, including regulation of tumor growth, microbial infections, cholesterol, cardiovascular diseases, diabetes, and metabolic syndrome^1^. These benefits are mainly due to its high content of alpha-linolenic acid, lignans, proteins, phenolic acids, and flavonoids, which enhance the body’s defense against carcinogens and contribute to its protective effects^2^. Flaxseed is also a rich source of essential nutrients like fat (mainly ALA), protein, fiber, and carbohydrates, making it a valuable addition to the diet^1,3^.

The increasing awareness of the link between diet and health has led to a growing interest in functional foods. Seeds and sprouts from various plants are considered functional foods due to their health benefits, including antioxidant, anticancer, antidiabetic, hypolipidemic, and anti-inflammatory properties. Germination enhances the nutraceutical properties of plants by activating hydrolytic enzymes, which release phenolic compounds from the cell wall into soluble forms^4–7^. Additionally, phenolic compounds are produced through metabolic pathways like the pentose phosphate, glycolytic, acetate/malonate, and hydrolytic tannin pathways, contributing to the diversity of these bioactive compounds^8–10^.

Beta-glucosidases (EC 3.2.1.21) are important enzymes in many living organisms, including fungi, bacteria, plants, and animals^11,12^. They are capable of breaking the β-glucosidic bonds of di-, oligosaccharide or other sugar conjugates and play important functions in many biological pathways, including cellulosic biomass degradation and secondary metabolite modification^13^. During germination, these enzymes cleavage the β-glucosidic bond in phenolic compounds, primarily conjugated to sugar residues bound to the hydroxyl group. This release boosts the level of free polyphenols, which can improve the plant’s health benefits and nutritional value^14^.

Reactive oxygen species (ROS) are critical in seed germination and dormancy regulation. While ROS production is a normal part of plant metabolism, environmental factors can cause excessive ROS production, which must be balanced for proper seed germination and dormancy regulation^15^. Enzymes such as polyphenol oxidase (PPO), peroxidase (POD), and catalase (CAT), along with non-enzymatic antioxidants like phenolic compounds, proline, and ascorbic acid, help scavenge ROS. CAT and POD convert hydrogen peroxide (H_2_O_2_) into water and oxygen, while PPO and POD oxidize phenols to quinone compounds, turning them into dark pigments^16^. Phenylalanine ammonialyase (PAL) is the first enzyme in phenylpropanoid metabolism, converting aromatic amino acids into trans-cinnamic acid, and its activity is induced during germination^17^. Studies show that flaxseed germination increases levels of fatty acids, vitamin C, phenolic compounds, and total antioxidant activity while decreasing lipid content during the first week^18,19^.

Although flaxseed is a rich source of bioactive compounds like lignans, phenolic acids, and flavonoids, their bioavailability is often limited by the seed’s structure and antinutritional factors^20^. While germination is known to improve the nutritional quality of seeds, no study has investigated how phenolic biosynthesis in flaxseed is activated through the action of its endogenous enzymes. Moreover, limited research has connected these biochemical changes to important functional properties such as antioxidant, antibacterial, and glucose-regulating activities. To address this gap, we used a two-level factorial design, which allows multiple factors and their interactions to be studied at the same time, unlike traditional one-factor-at-a-time method^21^. This approach provides a more complete understanding of how germination and enzymatic activity together enhance phenolic content and its related health benefits.

To address these gaps, the current study aimed to enhance the nutritional and functional properties of flaxseeds through controlled germination by:

(1) Evaluating the effects of germination on total phenolic content (TPC), total flavonoid content (TFC), and antioxidant activity. (2) Investigating the role of specific endogenous enzymes, including β-glucosidase, peroxidase, polyphenol oxidase, and catalase, in modulating these bioactive compounds. (3) Developing a statistical model based on a two-level factorial design, using enzyme activity as input variables to improve total phenolic content (TPC) and total flavonoid content (TFC) biosynthesis during germination. (4) Assessing the resulting biological activities of germinated flaxseed, including antioxidant, antibacterial, and glucose-regulating properties, to support its potential in functional food applications.

This study introduces a novel approach by modeling key endogenous enzymes as independent variables in a factorial model to uncover their mechanistic roles and synergistic interactions in phenolic biosynthesis during germination. Unlike the traditional approaches that consider these enzymes as observed outcomes, this strategy provides a clearer understanding of how enzyme activity drives the enhancement of phenolic compounds. It also offers a validated and practical framework for improving the functional quality of flaxseed through targeted germination processes.

## Materials and methods

### Seed germination

The seeds of Egyptian flax (*Linum usitatissimum* L.) were collected and identified by the Botany Department at the Agricultural Research Centre (ARC) in Giza, Egypt. The flax seeds (10 g) were surface-sterilized for 5 min with a 0.1% NaClO solution, then rinsed for 15 min with sterile distilled water. The seeds were placed on filter paper in Petri dishes (5 cm in diameter) and incubated at 25 ± 5 °C in the dark. Sprouts were collected daily over 8 days for analysis and stored at − 20 °C^8^.

### Preparation of flaxseed extracts

Ten grams of seeds or sprouts were dried overnight in an oven at 50 °C. The dried material was extracted with 20 ml of 80% methanol (v/v) at 25 °C under agitation (150 rpm) overnight. The extraction mixture was then filtered using Whatman No. 1 filter paper. The filtrate was centrifuged at 4000 rpm for 10 min at 4 °C. After centrifugation, the supernatant was collected, and the solvent was evaporated^8^.

### Determination of total phenolic and flavonoid concentrations

The total phenolic content (TPC) was determined as described by Velioglu et al.^22^. The reaction mixture contained 50 µl of the methanol extract, 100 µl of Folin–Ciocalteu reagent, and 850 µl of distilled water, and was allowed to stand at room temperature for 5 min. Then, 500 µl of 20% sodium carbonate solution was added, and the mixture was incubated at room temperature for 30 min. The absorbance was measured at 750 nm. Total phenolic content was calculated using a calibration curve prepared with known concentrations of gallic acid and expressed as milligrams of gallic acid equivalents (GAE) per gram of dry weight (DW).

The total flavonoid content (TFC) of the extracts was assessed using a colorimetric method described by Zhishen et al. with slight modifications^23^. Briefly, 250 µl of the methanol extract was mixed with 1.25 ml of distilled water and 75 µl of 5% NaNO_2_ solution. After 6 min, 150 µl of 10% AlCl₂ solution was added. One minute later, 0.5 ml of 1 M NaOH and 275 µl of distilled water were added, and the mixture was thoroughly mixed. The absorbance was measured at 510 nm. A standard curve was constructed using catechin in the range of 5–300 mg/kg, and results were expressed as milligrams of catechin equivalents per gram of dry weight (mg CE/g DW).

### HPLC analysis

HPLC analysis was conducted using an Agilent 1100 system with a C18 column and a diode-array detector, following the method of Kim et al.^24^. A gradient elution of acetonitrile and 2% acetic acid in water was run over 70 min at 1.0 ml/min. The methanol extracts were filtered before being injected (10 µl). Phenolic compounds were identified based on their retention times and UV spectra, which were compared to standard phenolic compounds at wavelengths of 280, 320, and 255 nm.

### Antioxidant activity (DPPH and ABTS Assays)

The antioxidant activity of methanolic flaxseed extracts was assessed using DPPH and ABTS radical scavenging assays^25,26^. For DPPH: 0.1 mL of extract was mixed with 0.9 mL of 0.1 mM DPPH in methanol, incubated in the dark for 30 min, and absorbance was measured at 517 nm. For ABTS: ABTS⁺ was generated by mixing 7 mM ABTS with 2.45 mM potassium persulfate (1:0.5), incubated for 12–16 h in the dark, then diluted to an absorbance of 0.70 ± 0.02 at 734 nm. A 0.1 mL extract was added to 0.9 mL ABTS solution, and absorbance was read after 1 min.

In both assays, $Scavengingactivity(\% )\, = \left[ {\left( {O.D.{\text{ }}control{\text{ - }}O.D.{\mkern 1mu} sample} \right)} \right]{\mkern 1mu} \, \times 100$

IC_50_ refers to the concentration of the phenolic extract required to inhibit 50% of DPPH or ABTS free radicals, and was determined by plotting the percentage of radical scavenging activity against varying concentrations of gallic acid equivalents (GAE) in the extract (data not shown).

### Determination of total antioxidant index

While IC_50_ values are commonly used to measure antioxidant potency, they do not reflect the total antioxidant activity per gram of sample. Therefore, a new calculation was proposed to determine total antioxidant index: Total antioxidant index = Total phenolic content (mg GAE/g DW)/mg IC_50_^10^. This method aligns with established recommendations for expressing antioxidant capacity in both concentration- and weight-based formats, and was supported by previous studies^8,12,27^.

### Determination of enzymatic activities

#### Crude enzyme extraction

One gram of flax seeds or sprouted seeds was homogenized with 20 mM Tris–HCl buffer (pH 7.2) using a glass homogenizer. The resulting homogenate was centrifuged at 13,500 × g for 10 min at 4 °C. The supernatant obtained was considered the crude extract and was stored at − 20 °C for subsequent analysis^8,10^.

#### Assay of PAL activity

The activity of phenylalanine ammonia-lyase (PAL, EC 4.3.1.24) was assessed following the method outlined by Goldson et al.^28^. The reaction mixture consisted of 0.1 ml of crude enzyme extract, 40 mM phenylalanine, and 20 mM Tris–HCl (pH 8.8). The mixture was incubated at 37 °C for 30 min. To terminate the reaction, 200 µl of 2% trichloroacetic acid (TCA) was added after incubation, and the samples were then centrifuged at 13,000 × g for 15 min. The absorbance at 290 nm was measured to quantify the amount of trans-cinnamic acid produced, which was determined using a standard curve.

#### *β*-GL activity assay

*β*-Glucosidase (β-GL) activity was measured as described by Gunata et al.^29^. The enzyme activity was assayed by incubating 0.9 mM p-nitrophenyl-β-D-glucopyranoside in 20 mM acetate buffer (pH 5.5) with 0.1 ml of the enzyme solution at 37 °C for 20 min. The reaction was terminated by adding 0.6 ml of 1 M sodium carbonate. The amount of *p*-nitrophenol released was quantified by measuring the absorbance at 405 nm. One unit of enzyme activity was defined as the amount of enzyme required to release 1 µmol of *p*-nitrophenol per minute.

#### POX assay

Peroxidase (POX) activity was measured according to Miranda et al.^30^. The reaction mixture consisted of 1 mL containing 8 mM H₂O₂, 40 mM guaiacol, 20 mM sodium acetate buffer (pH 5.5), and 0.1 mL of crude extract. The variation in absorbance at 470 nm, resulting from the oxidation of guaiacol, was monitored for 1 min using a spectrophotometer at ambient temperature. One unit of peroxidase activity is defined as the amount of enzyme that causes an increase of 1.0 OD per minute under the specified assay conditions. The activity is expressed in U/g seeds.

#### CAT assay

Catalase (CAT) activity was quantified using the method described by Bergmeyer^31^. A substrate solution consisting of 25 mM H₂O₂ in 75 mM sodium phosphate buffer (pH 7.0) was prepared, and 1 ml of this solution was combined with 0.1 ml of crude extract. The reduction in absorbance at 240 nm was monitored for 1 min using a spectrophotometer at ambient temperature. One unit of enzyme activity is defined as the amount of enzyme that induces a change of 0.1 absorbance units per minute under the specified assay conditions. The activity is expressed in U/g seeds.

#### PPO assay

Polyphenol oxidase (PPO) activity was measured according to Jiang et al.^32^. A crude enzyme extract (0.1 ml) was quickly added to a 0.02 M catechol solution, prepared in a 0.01 M sodium phosphate buffer (pH 6.8). The change in absorbance was measured at 400 nm and room temperature. One unit of PPO activity is defined as the amount of enzyme that causes an increase of 0.1 in absorbance (O.D.) per minute under the specified assay conditions. The activity is expressed in U/g seeds.

### Anti-bacterial properties

The antibacterial activity of phenolic extracts from flaxseeds and 5-day sprouts was evaluated against *Escherichia coli* and *Staphylococcus aureus* using the agar well diffusion method^33^. Bacterial suspensions (10⁸ CFU/ml) were spread on Mueller–Hinton agar, and 50 µg GAE of each extract was added to wells, followed by incubation at 37 ± 1 °C for 18 h. Inhibition zones were measured to assess activity, with gentamicin as a control. The minimum inhibitory concentration (MIC) was determined via agar dilution, identifying the lowest extract concentration that inhibited visible bacterial growth after 18 h at 37 ± 1 °C.

### Anti-hyperglycemic properties

The in vitro anti-hyperglycemic potential of phenolic extracts from flaxseeds and 5-day sprouts was assessed through α-amylase and α-glucosidase inhibition assays, following the methods of Liu et al.^34^ and Zhang et al.^35^, respectively. For α-amylase inhibition, a reaction mixture containing 5 U of pancreatic α-amylase, sodium phosphate buffer (pH 7.2), and either the extract or acarbose was incubated at 37 °C, followed by starch addition, and absorbance was measured at 540 nm after color development with dinitro-salicylic reagent. α-Glucosidase inhibition was measured using one unit of enzyme with p-nitrophenyl-α-glucopyranoside as substrate in a buffer at pH 6.8, with absorbance read at 405 nm. In both assays, the percentage of enzyme inhibition was calculated based on the decrease in optical density, and the IC_50_ value represented the extract concentration required to inhibit 50% of the enzyme activity.

### Experimental design

To investigate the factors influencing the release of total phenolic content (TPC) and total flavonoid content (TFC) during flaxseed germination, a two-level factorial design was employed using Design-Expert^®^ Software Version 11 (Stat-Ease Inc., Minneapolis, MN, USA). This design was developed based on findings from preliminary single-variable experiments, which helped identify the most influential enzymatic and germination-related factors. Six independent variables were selected based on their known roles in phenolic biosynthesis and metabolism: germination day (GD), β-glucosidase (β-GL), phenylalanine ammonia-lyase (PAL), peroxidase (POX), polyphenol oxidase (PPO), and catalase (CAT). Each factor was evaluated at two levels (low and high) to assess its effect on TPC and TFC production. The experimental design consisted of 14 runs, including 7 factorial combinations replicated in two batches. Each treatment was performed in triplicate to ensure statistical reliability. The data were analyzed using factorial modeling to evaluate both the main effects and interactions between the variables. An empirical model was generated to describe the relationship between the input variables and the measured responses (TPC and TFC), allowing for the identification of significant and synergistic effects.

An empirical relationship between the input parameters and the output responses was established using regression analysis, as shown below:

### TPC equations

#### In terms of actual factors

$$\begin{gathered} {\text{TPC }} = {\text{ }} + {\text{6}}.{\text{424 }} - {\text{ 2}}.{\text{315 }} \times \,{\text{GD }} + {\text{ }}0.{\text{348}}\, \times \,\beta - {\text{GL }} - {\text{ }}0.{\text{882}}\,\, \times \,{\text{PAL }} + 0.0{\text{2}}0\, \hfill \\ \times {\text{POX }} + {\text{ }}0.0{\text{22}} \times {\text{PPO }} + {\text{ }}0.0{\text{4}}0{\text{2}} \times {\text{CAT }} + {\text{ }}0.{\text{136}} \times {\text{ }}\left( {{\text{GD }} \times {\text{ }}\beta - {\text{GL}}} \right){\text{ }} \hfill \\ - {\text{ }}0.00{\text{4}}\beta - {\text{GL}} \times {\text{PPO}} + {\text{ }}0.00{\text{4}} \times {\text{ }}\left( {{\text{PAL }} \times {\text{ PPO}}} \right) \hfill \\ \end{gathered}$$

### In terms of coded factors

$$\begin{gathered} {\text{TPC }} = {\text{ }} + {\text{1}}0.{\text{18 }} + {\text{5}}.{\text{51 A }} + {\text{ 2}}.{\text{35 B }} - {\text{1}}.{\text{6}}0{\text{ C }} + {\text{2}}.{\text{38 D }} - {\text{2}}.{\text{67 E }} \hfill \\ + {\text{1}}.{\text{54 F }} + {\text{4}}.{\text{41 AB }} - {\text{5}}.{\text{83 BE }} + {\text{5}}.{\text{81 CE}} \hfill \\ \end{gathered}$$

### TFC equations

#### In terms of actual factors

$$\begin{gathered} {\text{TFC }} = {\text{ }} - {\text{1}}.0{\text{11 }} + {\text{ }}0.{\text{182}} \times {\text{GD }} - {\text{ }}0.0{\text{27}} \times \beta - {\text{GL }} - {\text{ }}0.0{\text{29}} \times {\text{PAL }} - {\text{ }}0.0{\text{165}} \times {\text{POX }} \hfill \\ - {\text{ }}0.0{\text{38}} \times {\text{PPO }} + {\text{ }}0.0{\text{45}} \times {\text{CAT }} + {\text{ }}0.00{\text{4}} \times \,\,\left( {{\text{GD }} \times {\text{ POX}}} \right){\text{ }} + {\text{ }}0.00{\text{1}} \times \hfill \\ {\text{ }}\left( {\beta - {\text{GL }} \times {\text{ PPO}}} \right){\text{ }} - {\text{ }}0.000{\text{72}} \times \left( {\beta - {\text{GL }} \times {\text{ CAT}}} \right) \hfill \\ \end{gathered}$$

#### In terms of coded factors

$$\begin{gathered} {\text{TFC }} = {\text{ }} + {\text{1}}.0{\text{7 }} + {\text{ 2}}.{\text{84 A }} + 0.{\text{9294 B }} - 0.{\text{2685 C }} + 0.{\text{5791 D }} - \hfill \\ 0.{\text{4177 E }} + 0.{\text{9388 F }} + {\text{1}}.{\text{97 AD }} + {\text{1}}.{\text{65 BE }} - 0.{\text{2566 BF}} \hfill \\ \end{gathered}$$

These models were used to identify significant contributors and interactions in the biosynthesis of phenolic and flavonoid compounds during germination.

### Statistical analysis

The data were analyzed using a one-way ANOVA, followed by Tukey’s post hoc test and correlation analysis, all performed with GraphPad Prism 5 software. Results are presented as means ± standard deviation (*n* = 4), with statistical significance at *P* < 0.01.

All experimental procedures were carried out in compliance with relevant guidelines.

## Result and discussion

### Enhanced total phenolic and flavonoid contents of flaxseeds during germination

Flaxseeds are considered a highly promising functional food due to their rich content of physiologically active phenolic compounds and flavonoids. The physiological roles of flaxseed polyphenols, including their antioxidant, anti-inflammatory, anti-atherosclerotic, and anti-cancer properties, have attracted increasing attention^36^. Therefore, the present study investigates the phenolic profile and antioxidant potential in dry and germinated flaxseeds. The total phenolic content (TPC) and total flavonoid content (TFC) in flaxseeds and sprouts during eight days of germination are shown in Table 1.

**Table 1:** Total phenolic and flavonoid contents of the flaxseeds during germination.

Days	Total phenolic mg GAE/g DW	Total flavonoid mg CE/g DW	CE/GAE (%)
0 (seeds)	1.9 ± 0.141^a^	0.087 ± 0.007^a^	4.55
1	3.6 ± 0.4^b^	0.305 ± 0.02^b^	11.8
2	6.8 ± 0.45^c^	0.88 ± 0.03^c^	13.0
3	8.7 ± 0.28^c^	1.32 ± 0.29^d^	15.0
4	11.3 ± 0.41^d^	2.2 ± 0.045^e^	19.4
5	14.5 ± 0.35^e^	3.33 ± 0.25^f^	22.9
6	10.3 ± 0.28^f^	1.8 ± 0.35^g^	17.4
7	8.6 ± 0.120^c^	1.27 ± 0.32^d^	14.7
8	7.2 ± 0.0.49^c^	0.89 ± 0.06^c^	12.3

GAE, gallic acid equivalent; CE, Catechin equivalent. Values are presented as means ± SD (*n* = 4). Values in the same column with different superscripts (a, b, c, d, e, f, g) are significantly different at (*P* < 0.01).

Both TPC and TFC significantly increased during the germination process, peaking on day five. The TPC increased from 1.9 mg GAE/g in dry seeds to 14.5 mg GAE/g in 5-day-old sprouts, reflecting a 7.6-fold increase. Similarly, TFC increased from 0.087 to 3.33 mg CE/g, marking a 38.27-fold rise in sprouted seeds. From 5-day to 8-day, the levels of both TPC and TFC gradually decreased, reaching 7.2 mg GAE/g and 0.89 mg CE/g, respectively. Additionally, the ratio of TFC to TPC showed a significant increase, rising from 4.55% in dry flaxseeds to a peak value of 22.9% on day five of sprouting.

Earlier research has shown that the types and amounts of phenolic compounds in dry flaxseed vary between different flaxseed varieties. The total phenolic content (TPC) found in the present study is similar to what was reported in previous research in Egypt (0.046–3.62 mg GAE/g)^37,38^. Alu’datt et al.^39^ found that the TPC ranged from 0.90 to 4.69 mg/g, and this difference was due to the different methods used to extract the compounds. In six varieties of Indian flaxseed, low phenolic content (0.062 to 0.085 GAE/g) was observed^40^, while Chinese flaxseed varieties had much higher levels, reaching 4.74 mg GAE/g^41^. Flavonoids are water-soluble polyphenolic compounds found widely in plants, often in the form of glycosides. They work by scavenging free radicals or binding to metals^42^. Flavonoids are a major component of the phenolic compounds in flaxseed, with concentrations ranging from 35 to 71 mg/100 g^3^.

An increase in the concentration of total phenolics and flavonoids was observed during the germination of different edible seeds and sprouts^19^. A similar pattern was reported in a study by Li et al. (2019)^43^. He found that the total phenolic content increased from 1.56 to 6.56 mg GAE/g in brown flaxseeds, and from 1.11 to 7.89 mg GAE/g in golden flaxseeds after 5 days of germination, showing increases of 4.2 and 7.1 times, respectively. Additionally, the TPC and TFC levels and their increasing trends during the first 5 days of germination align with findings reported by Liu et al.^44^ and Huang et al.^18^.

From day 5 to day 8 of germination, the decline in total phenolic and flavonoid content in flaxseed sprouts may result from multiple physiological processes. As germination advances, phenolic compounds may be consumed as antioxidants to mitigate oxidative stress or degraded by oxidative enzymes. Additionally, these compounds might be transformed into bound forms or redirected toward lignin synthesis and cell wall formation^45–47^.

In this study, HPLC analysis revealed that both dry and 5-day sprouts contained 11 phenolic acids (Table 2) and Fig S1. For 5-day sprouts, the levels of these compounds increased significantly, showing 2.4- to 6.4-fold rises. Previous research has shown that flaxseed contains a wide range of phenolic compounds. Besides lignans, flaxseeds include 8–10 g/kg of phenolic acids, mainly p-coumaric, vanillic, sinapic, and ferulic acids, which are typically present as glycosides bound by ester and ether linkages^36,48^. HPLC analysis by Yaqoob^49^ also identified several key compounds in flaxseed ethanolic extracts, such as gallic acid, secoisolariciresinol diglucoside, caffeic acid, coumaric acid, hydroxybenzoic acid, ferulic acid, benzoic acid, kaempferol, and cinnamic acid. The observed increase in free phenolic compounds during germination is likely due to the release of cell wall-bound phenolics, which enhances their concentration in the sprouted seeds^50^.

**Table 2:** Phenolic compounds of the flax dry seeds and 5-day sprouts using HPLC analysis.

Compound	Dry seeds mg/g DW	Day 5 sprouts mg/g DW	A fold increase in sprout
Gallic	0.15^a^	0.92^b^	6.1
*p*-hydroxybenzoic	0.20^a^	0.72^b^	3.6
Cateachin	0.18^a^	0.81^b^	4.5
Caffeic	0.25^a^	1.2^b^	4.8
Syringic	0.17^a^	0.62^b^	3.6
Vanillic	0.05^a^	0.24^b^	4.8
Ferulic	0.35^a^	1.37^b^	3.9
Sinapic	0.20^a^	1.29^b^	6.4
*p*-coumaric	0.40^a^	2.2^b^	5.5
Rosmarinic	0.23^a^	0.9^b^	3.9
Cinnamic	0.10^a^	0.24^b^	2.4

RT: Retention Time. Values are presented as means ± SD (*n* = 4), Values in the same raw with different superscripts (a and b) are significantly different at (*P* < 0.01).

### Improved antioxidant activity in flaxseed during germination

Phenolic compounds play a crucial role in antioxidant activity, largely due to the positioning of hydroxyl groups on their aromatic rings, which is closely linked to the antioxidant properties of phenolic acids^48^. DPPH and ABTS are stable free radicals that interact with antioxidant substances and readily accept hydrogen atoms^51^. During seed germination, the concentration of phenolic compounds increased, potentially enhancing the seeds’ ability to scavenge free radicals. In this study, the antioxidant properties of both raw and germinated seeds were evaluated by analyzing the effectiveness of methanolic seed extracts in neutralizing DPPH and ABTS radical cations, as displayed in Tables 3 and 4. The scavenging activities of DPPH and ABTS were higher in germinated seeds compared to raw seeds. Notably, 5-day sprouts demonstrated significantly enhanced radical quenching abilities, with IC_50_ values of 0.0082 and 0.0036 mg GAE/mL for DPPH and ABTS, respectively. These values reflect approximately 2.26- and 2.6-fold increases in scavenging activity compared to raw seeds, which exhibited IC_50_ values of 0.0082 and 0.0186 mg GAE/ml. Since a lower IC_50_ indicates stronger radical scavenging capacity, these results highlight the enhanced antioxidant potential following germination. However, the IC_50_ values for both radicals increased by the eighth day of germination, reaching 0.0135 and 0.0052 mg GAE/mL for DPPH and ABTS, respectively.

**Table 3:** The antioxidant activity of the flaxseeds during germination days was determined using the DPPH assay.

Days	IC_50_ (mg GAE/ml)	Total antioxidant index (Total phenolic content g DW/IC_50_)
0 (seeds)	0.0186 ± 0.001^a^	102 ± 8.3^a^
1	0.0165 ± 0.001^b^	218 ± 13^b^
2	0.0138 ± 0.0008^b^	492 ± 24^b^
3	0.0118 ± 0.00071^c^	737 ± 30^c^
4	0.0100 ± 0.0006^c^	1130 ± 77^c^
5	0.0082 ± 0.0003^d^	1768 ± 85^d^
6	0.0092 ± 0.0004^c^	1119 ± 82^c^
7	0.0115 ± 0.008^b^	747 ± 33^c^
8	0.0135 ± 0.001^b^	533 ± 22^b^

IC_50_ value is the phenolic concentration required to scavenge 50% of DPPH. Values are presented as means ± SD (*n* = 4), Values in the same column with different superscripts (a, b, c, d) are significantly different at (*P* < 0.01).

**Table 4:** The antioxidant activity of the flaxseeds during germination days was determined using the ABTS assay.

Days	IC_50_ (mg GAE/ml)	Total antioxidant index (Total phenolic content g DW/IC_50_)
(seeds) 0	0.009 ± 0.0006^a^	211 ± 8.51^a^
1	0.0084 ± 0.0006^b^	428 ± 19^b^
2	0.0072 ± 0.0005^b^	944 ± 22^b^
3	0. 0064 ± 0.0004^c^	1359 ± 88^c^
4	0.0044 ± 0.0003^c^	2568 ± 122^c^
5	0.0036 ± 0.0003^d^	4027 ± 142^d^
6	0.004 ± 0.00032^c^	2575 ± 111^c^
7	0.0046 ± 0.0004^c^	1869 ± 99^c^
8	0.0052 ± 0.0003^b^	1384 ± 78^b^

The IC_50_ value is the phenolic concentration required to scavenge 50% of ABTS. Values are presented as means ± SD (*n* = 4). Values in the same column with different superscripts (a, b, c, d) are significantly different at (*P* < 0.01).

A novel calculation method was developed to assess total antioxidant activity accurately by incorporating total phenolic content (TPC) and IC_50_ values from DPPH and ABTS radical assays. The formula used is: Total antioxidant index = TPC (mg GAE/g DW) / IC_50_^10^. Results obtained using this method mirrored the trends observed in DPPH and ABTS scavenging activities during germination, as shown in Tables 3 and 4. During germination, the TPC/IC_50_ ratios for both radicals increased markedly in sprouts, reaching peak values on 5-day sprouts (1768 for DPPH (17.3-fold increase) and 4027 for ABTS (19.1-fold increase)) compared to raw seeds. After this peak, total antioxidant index gradually declined, with values dropping to 533 and 1384 for DPPH and ABTS, respectively, at day 8.

### Correlation of phenolic compounds with antioxidant activity during seed germination

**Fig. 1:**
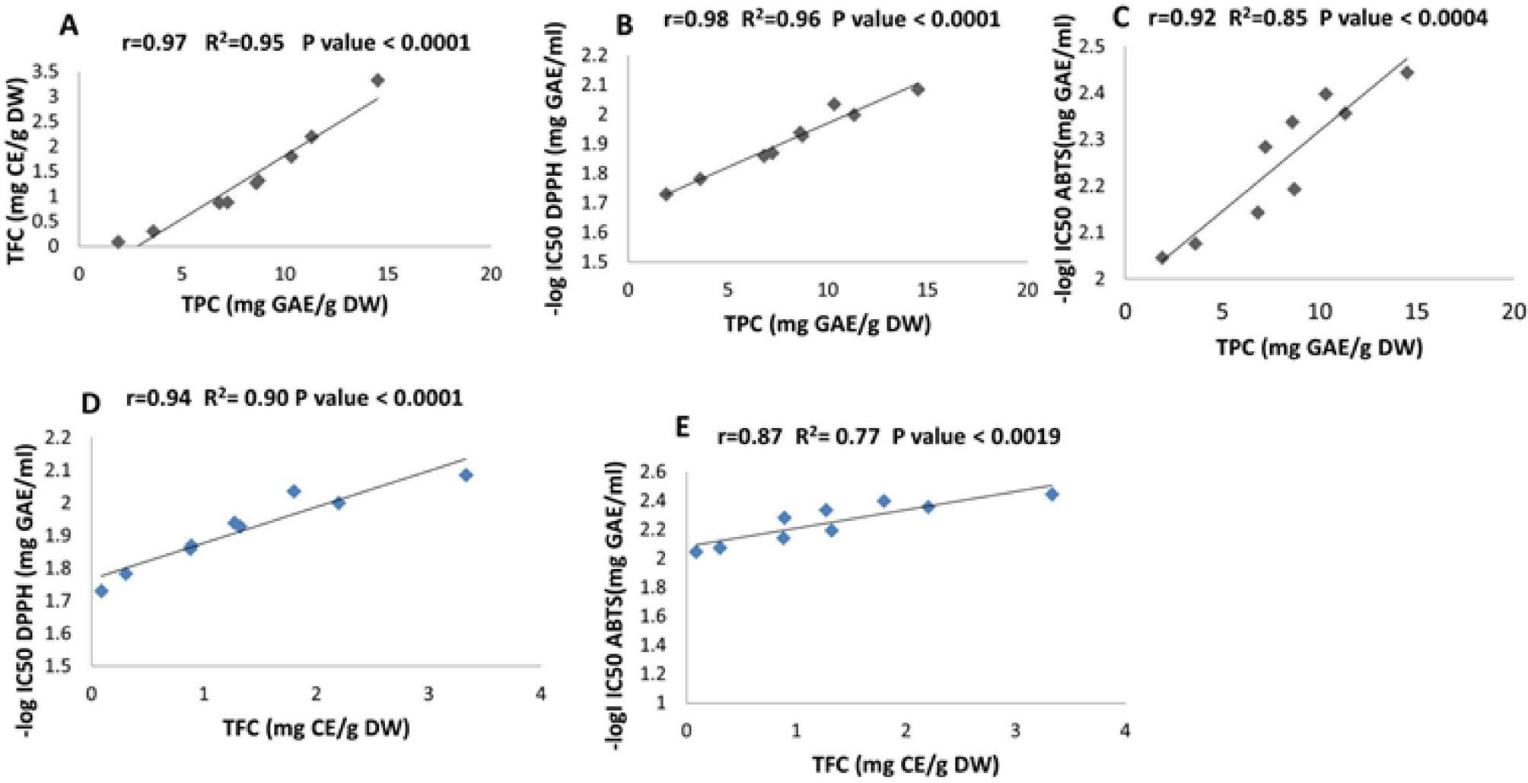
A scatter plot illustrating the Pearson correlation for the relationships among the following variables during germination using Prism software: (**A**) TPC and TFC, (**B**) TPC and –log IC_50_ DPPH, (**C**) TPC and –log IC_50_ ABTS, (**D**) TFC and –log IC_50_ DPPH, and (**E**) TFC and –log IC_50_ ABTS.

During germination, seeds transition from dormancy to active growth, triggering intense metabolic activity that generates reactive oxygen species (ROS) and results in oxidative stress. To counter this, plants activate both enzymatic and non-enzymatic antioxidant defense systems. The enzymatic defenses involve the upregulation of key enzymes such as catalase (CAT) and peroxidase (POD), which help neutralize ROS and protect cellular components. The non-enzymatic defenses include activation of the phenylpropanoid pathway, resulting in increased biosynthesis of phenolic compounds, flavonoids, and other antioxidant secondary metabolites. Here, the relationships between total phenolic content (TPC), total flavonoid content (TFC), and the scavenging activities of DPPH and ABTS were analyzed using Prism software, as illustrated in Fig. 1A–C. All correlations were strong and statistically significant. The Pearson correlation coefficient (r) and the coefficient of determination (R²) exhibited the highest values between TPC and DPPH activity, with *r* = 0.98 and R² = 0.96. This was followed by the relationship between TPC and TFC, which showed *r* = 0.97 and R² = 0.95, and lastly, the correlation between TPC and ABTS activity, where *r* = 0.92 and R² = 0.85. The correlation between TFC and DPPH and ABTS is shown in Fig. 1D and E, respectively. Figure 1D showed a high correlation (*r* = 0.946, R2 = 0.90), while Fig. 1E showed a comparatively lower correlation (*r* = 0.87, R2 = 0.77). Previous studies have consistently shown that radical scavenging activity is closely aligned with the phenolic content of plant extracts. For example, the antioxidant activity in extracts of black chokeberry and blueberry has been attributed to their high total phenolic content^52^. Similarly, a strong relationship between TPC and antioxidant activity has been reported across various plant extracts^53^. Zhou et al. further demonstrated a significant positive correlation between TFC and free radical scavenging activity against both DPPH and ABTS radicals^54^. In the case of *Curcuma longa*, Pearson correlation analysis revealed that both TPC and TFC significantly influenced antioxidant activity^55^. A strong association between antioxidant activity and TPC was also observed in germinated peanut extracts, highlighting phenolic compounds as the primary contributors to antioxidant capacity in those sprouts^56^.

Recent studies confirm that germination significantly enhances antioxidant activity through increased phenolic biosynthesis. Chia seeds showed a 6–11-fold rise in phenolic and flavonoid content and up to 29-fold higher antioxidant activity after 7 days^8^. Wild mustard sprouts exhibited 5–10-fold increases in phenolics and up to 21-fold stronger antioxidant capacity^57^. Broccoli and wheat also demonstrated marked improvements. A study on 17 edible seeds, including white radish, linked germination to activation of the phenylpropanoid pathway and substantial increases in antioxidant properties^58^. In line with these findings, the present study concludes that germination markedly enhanced the antioxidant properties of flax seeds, and correlation analysis confirmed that both TPC and TFC are key factors contributing to the increased radical scavenging activity during germination.

### Modulation of enzyme activities throughout germination

**Fig. 2:**
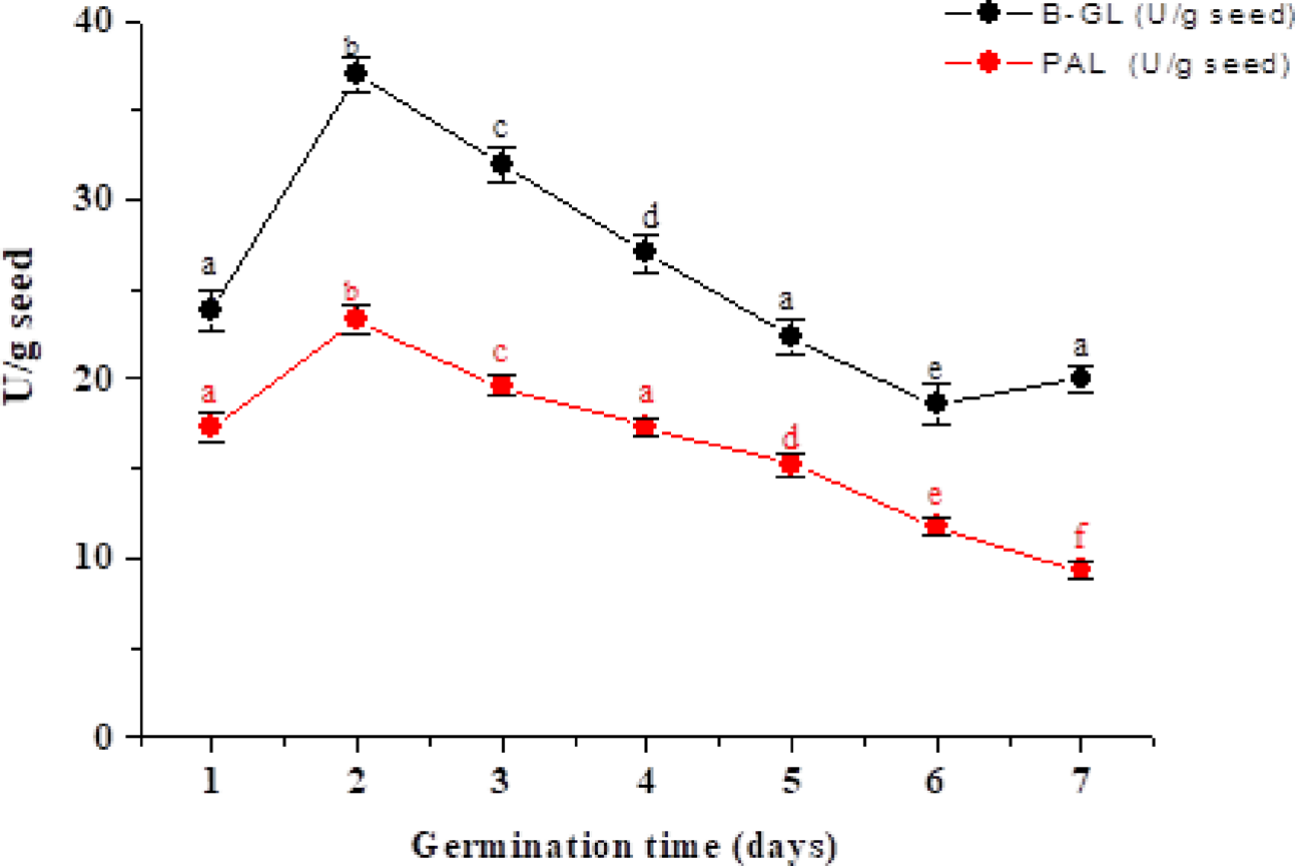
Screening of the enzymatic activities of the *β*-glucosidase (β-GL) and phenylalanine ammonia-lyase (PAL) as carbohydrate-cleaving and phenolic-synthesizing enzymes during the germination of flaxseeds, respectively. Points are presented as means ± SD (*n* = 4). Values with different superscript letters within the same enzyme indicate significant differences at *P* < 0.01.

Germination triggers substantial alterations in the phenolic composition of seeds, primarily due to the activation of endogenous enzymes and complex metabolic processes^59^. A notable increase in total phenolic content (TPC) during this stage is attributed to the mobilization and redistribution of phenolic acids, facilitated by the enzymatic hydrolysis of starch reserves^60^. Phenylalanine ammonialyase (PAL), a key enzyme in the phenylpropanoid pathway, catalyzes the conversion of L-phenylalanine, derived from the shikimic acid pathway, into trans-cinnamic acid by removing an ammonia group. This intermediate is further transformed into a series of compounds such as coumaric, asafetida, and sinapic acids, which serve as precursors for the synthesis of diverse secondary metabolites, including lignin, flavonoids, chlorogenic acid, and CoA esters^61^. Additionally, β-glucosidase (β-GL) has been identified as a crucial enzyme in the biosynthesis of major phenolic compounds, particularly in fruits such as virgin olive, where it contributes to the release of bound phenolics^62^. This study found β-GL and PAL activities significantly increased within the first two days of germination, peaking at 36.96 and 23.34 U/g, respectively. After day 2, β-GL levels declined gradually, while PAL activity decreased more rapidly, reaching 20 and 9.37 U/g by day 7 (Fig. 2). These findings align with previous research; for instance, a notable rise in β-GL activity was recorded in soybean seeds during a 72-hour germination period^14^. Conversely, Akiyama et al.^63^ observed that β-GL activity in rice seeds increased over eightfold within five days of germination. A clear positive correlation was observed between PAL activity and the accumulation of phenolic compounds in buckwheat sprouts^17^. PAL enhances seed vigor and promotes seed germination under high-temperature, salt, and drought stress by increasing antioxidant enzyme activity and flavonoid accumulation while reducing oxidative damage^64^.

### Progressive changes in PPO, POX, and CAT activities during flaxseed germination

**Fig. 3:**
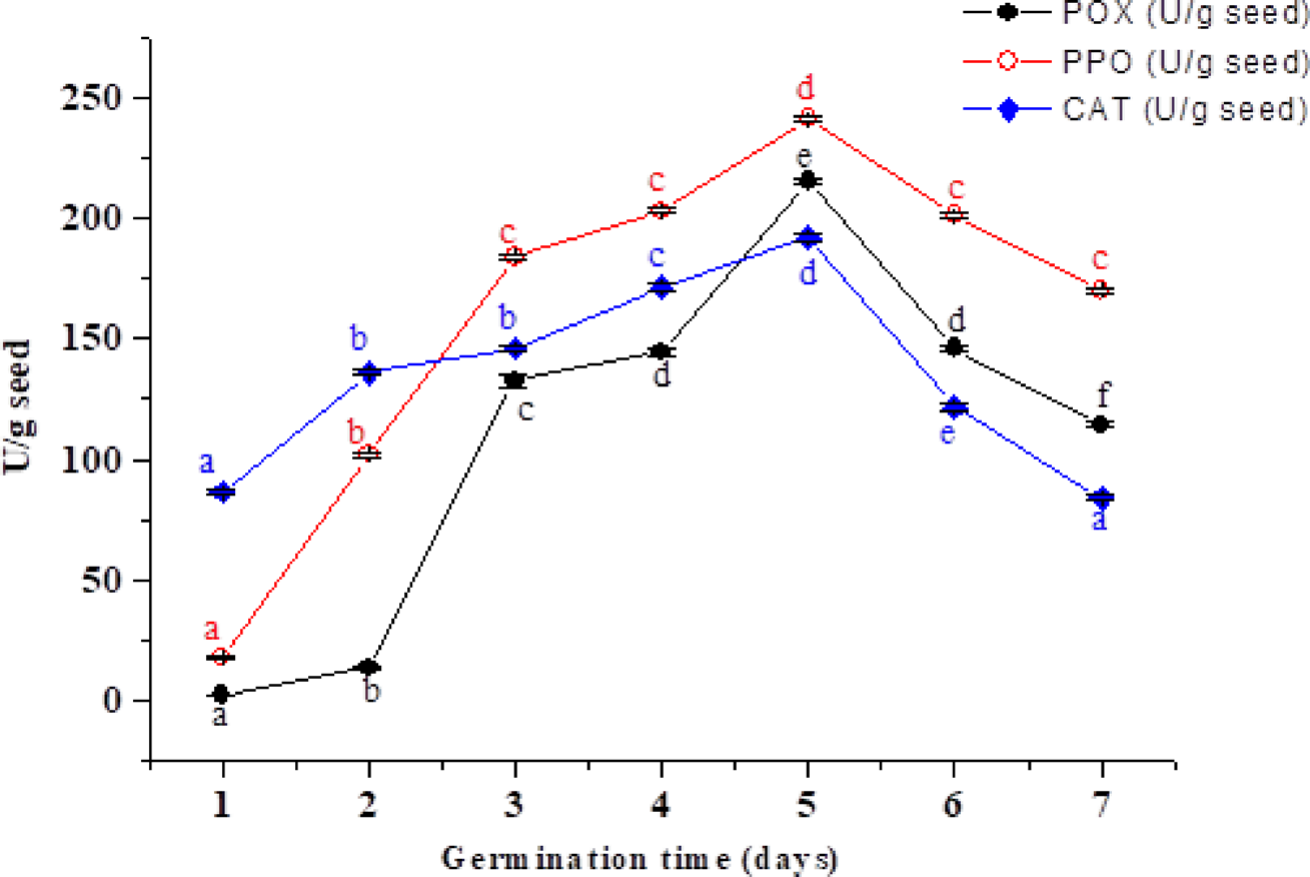
Screening of the enzymatic activities of the polyphenol-oxidase (PPO), catalase (CAT), and peroxidase (POX) as antioxidant enzymes during the germination of flax seeds. Points are presented as means ± SD (*n* = 4). Values with different superscript letters within the same enzyme indicate significant differences at *P* < 0.01.

Seed germination marks the beginning of a plant’s life cycle, involving tightly regulated cellular and metabolic activation. As germination progresses and mitochondrial function resumes, increased oxygen uptake enhances oxidative phosphorylation, potentially leading to oxidative stress^65^. Effective detoxification is crucial for seed viability, desiccation tolerance, and successful germination^66^. Enzymes like polyphenol oxidase (PPO) help mitigate stress by producing quinones from phenolic compounds^67^, while catalase (CAT) plays a vital role in early germination, particularly in oily seeds, by breaking down hydrogen peroxide generated during fatty acid β-oxidation^68^. This study examined the activities of PPO, POX, and CAT enzymes during flax seed germination (Fig. 3), showing a progressive increase that peaked on day 5, with PPO, POX, and CAT rising 13.56-fold (240.66 U/g), 103.11-fold (215.5 U/g), and 2.23-fold (192.13 U/g), respectively, compared to dry seeds. Enzyme activities then declined slightly over the following two days. These findings are consistent with the results of Salah et al.^57^, who observed peak POX activity and antioxidant potential in 5-day mustard sprouts, as well as with prior studies on garden cress^10^ and maize^69^, where POX, CAT, and PPO activities similarly peaked between days 4–6 of germination.

### Statistical modeling of phytochemical biosynthesis with endogenous enzymes as key variables

#### Statistical analysis and model fit summary

This study uses a two-level full factorial design in Design-Expert^®^ software to explore interactions between various parameters during flaxseed germination. It identifies key factors, such as germination days and endogenous enzymes, that influence TPC and TFC production. The study also estimates optimal factors for maximizing polyphenol release, enhancing nutritional value. Using the Minimum-Run Resolution V Screening Design (MRR-V) minimizes experimental runs, reducing costs and ensuring reliable results^70^. By understanding endogenous variable interactions, the study helps optimize factors for phytochemical biosynthesis, ultimately improving flaxseed nutritional quality.

In this study, antioxidant enzymes (POX, PPO, CAT) were included as input variables in the factorial model based on preliminary findings linking their activity to increased TPC and TFC during germination. Although typically treated as outcomes, these enzymes were modeled to explore their functional role in phenolic biosynthesis, as supported by previous research^71–73^. The screening study tested six parameters—GD (A), β-GL (B), PAL (C), POX (D), PPO (E), and CAT (F)—at two levels (low and high), resulting in 7 preliminary experiments replicated twice for a total of 14 runs, with each value being the mean of triplicate tests. The responses/products were TPC and TFC. ANOVA and fit statistics shown in Table S1 confirmed the statistical significance of the model, with F-values of 838.94 for TPC and 1124.79 for TFC. A p-value below 0.05 indicates significance, and the model demonstrated high consistency with adjusted R² values of 0.9983 and 0.9987, and predicted R² values of 0.992 and 0.991. Adeq Precision evaluates the signal-to-noise ratio, with a preference for ratios exceeding 4^74^. The Adeq. Precision ratios of 95.09 for TPC and 106.60 for TFC, both well above the threshold of 4, support the model’s reliability for exploring the design space.

#### Diagnostic plots

The diagnostic plots of expected vs. actual values (Fig. S2) confirm the adequacy of the model, showing a strong correlation between experimental and predicted data for both TPC and TFC. The alignment of experimental data points with the 45-degree line in Fig. S2 indicates a linear relationship, further supporting the model’s validity. Fig. S3 and Table S2 identify key factors influencing TPC and TFC development, using half-normal plots, Pareto charts, and regression analysis. For TPC: POX, GD, and β-GL are the primary factors, while for TFC: CAT, GD, POX, and β-GL are most significant with low value of P (< 0.05) and high value of F. The half-normal plots show that deviations from the red line indicate a strong model impact. POX had the most significant impact on TPC release, whereas CAT showed a smaller yet noticeable effect. In contrast, CAT exerted the strongest influence on TFC accumulation.

The goal of the optimization is to attain the highest yield of TPC and TFC. This can be effectively accomplished by modifying design parameters through the application of a suitable numerical optimization method that utilizes the desirability function approach. This method seeks to optimize multiple equations concurrently by transforming a multiple response issue into a singular one. Moreover, the desirability serves as an objective function that varies from zero beyond the limits to one at the target^75^. In the optimization process, all input parameters were chosen for optimization, along with their respective ranges (lower and upper limits of experimental data). Additionally, TPC and TFC were verified using maximized tools. The program identified a total of 100 solutions from which it recommended the optimal one, demonstrating that the maximum desirability achieved is 1 (Fig S4). Fig. S5 shows ramp function graphs, where the peaks indicate optimal settings. Red dots mark the optimized input values: GD (5.044 days), β-GL (37.1), PAL (16.05), POX (241.09), PPO (186.6), and CAT (101.84 U/g seeds). Blue dots represent the predicted optimal outputs: TPC (14.7 mg GAE/g) and TFC (3.55 mg CE/g). Table S3 shows that the predicted TPC and TFC values closely matched the experimental results, with small errors of 1.38% and 6.6%, respectively, confirming the model’s accuracy. On day 5 of germination, the concentrations of endogenous enzymes, β-GL (22.27 U/g), PAL (15.25 U/g), POX (215.5 U/g), PPO (240.66 U/g), and CAT (192.13 U/g), were identified as optimal for maximizing the biosynthesis of both TPC (14.5 mg GAE/g) and TFC (3.33 mg CE/g). These experimental results closely matched the predicted values obtained from the statistical model, confirming its accuracy and reliability (Table S3).

Table S2 also identifies the significant factor interactions for TPC and TFC biosynthesis, including GD × β-GL (AB), and PAL × PPO (CE) for TPC, and GD × POX (AD) and β-GL × PPO (BE) for TFC with low value of P (< 0.05) and high value of F. Interactions are classified as synergistic (positive coefficient) or antagonistic (negative coefficient)^76^. Synergistic effects were observed in interactions AB and CE for TPC, and AD and BE for TFC, indicating that the combined action of these factors enhanced phenolic and flavonoid release beyond the expected additive effects. Conversely, interaction BE showed an antagonistic effect on TPC. These findings demonstrate that specific enzyme combinations significantly improve bioactive compound biosynthesis, likely through complementary mechanisms in the germinated flaxseed matrix. GD positively impacts TPC and TFC in legume seeds, as confirmed by Borges-Martínez et al.^77^. During germination, β-GL breaks down glycoside conjugates and oligosaccharides from phenolic compounds, releasing sugars and aglycones, such as isoflavones in soybeans. Germinated soybeans contain β-GL, which enhances soy-based products by increasing bio-accessible phenolic compounds^78^. This process explains the positive link between β-GL and TPC/TFC during germination. Phenolic compounds are oxidized and generated by PPO, CAT, and POX during germination, with POX playing a key role in polyphenol synthesis and antioxidant activation^47^. Additionally, POX aids in lignin production by converting free phenolic acids into dimeric forms^79^. A positive correlation between POX, TPC, and TFC was observed, which is consistent with previous research on garden cress sprouts^57^. The increase in PAL levels influences phenolic compound production, though its activity can be inhibited by phenolic acids like ferulic and p-coumaric acids, which negatively affect TPC and TFC^80^. The relationship between CAT and flavonoids is in line with Zhang et al.^81^, and the direct link between POX, β-GL, and GAT with TPC and TFC underscores the significance of these enzymes during germination.

We use the numerical optimization of design expert software to further confirm the interaction and quantifying the synergistic effects of the interacting terms on TPC and TFC. We tested the different concentrations of the interacting factors (Fig S4) especially given maximum yield of TPC and TFC in the optimization approach in Table S3. Table S4 shows GD × β-GL (AB) and PAL × PPO (CE) interaction effect on TPC. In this technique the untested factors were set at minimum levels. To neglect the effect of β-GL we set it equal zero and GD at low value. The program gave best solution providing TPC of 1.64 mg GAE/g with desirability function of 0.84. At germination day zero and β-GL at minimum level the TPC was 1.25 with desirability of 1. At minimal levels of A and B the TFC yielded 3 mg GAE/g. TPC enhanced by increasing A or B to maximum values. At ultimate values of A (5 day) and B (37.8 U/g seed) TPC reached to 7.2 mg GAE/g. This result refers to the interaction between A and B has synergetic effect on TPC. This Phenomenon was also occurred in the interaction of PAL with PPO (CE). Individual C and individual E yielded low level of phenolic content. Increases in both can enhance TPC to 9.9 mg GAE/g. This result demonstrated a significant synergistic interaction between factors A and B; and C and E where their combined effect on the TPC was greater than the sum of their individual effects. This suggests that there is a cooperative effect between the factors that leads to an increase in TPC. Table S4 also illustrates synergetic effect of the interaction terms GD × POX (AD) and β-GL × PPO (BE) on TFC. TFC level changes when two combinations are changed accordingly.

Overall, the model effectively identified and refined the key factors, germination days (GD), β-glucosidase (β-GL), peroxidase (POX), and catalase (CAT), that significantly enhance TPC and TFC levels in germinated flaxseed. Statistical analysis using Design-Expert^®^ software confirmed the model’s reliability and accuracy, highlighting both individual and synergistic effects of these variables. This provides a validated strategy for enhancing the nutritional value of flaxseed through targeted germination conditions.

#### Enhanced antibacterial activity of germinated flaxseed

The antibacterial activity of dry and 5-day germinated flaxseed extracts was evaluated using inhibition zones and minimum inhibitory concentrations (MICs), with gentamicin as a standard (Table 5). The 5-day sprout extract showed significantly greater antibacterial effects against both *E. coli* and *S. aureus* compared to the raw-seeds extract. Inhibition zones increased from 5 mm (dry seeds) to 13 mm and 14 mm for *E. coli* and *S. aureus*, respectively as seen in Table 5 and Fig S6. MIC values confirmed this enhanced activity, with the sprout extract exhibiting a lower MIC (0.103 and 0.103 mg/mL) than the dry seed extract (0.32 and 0.41 mg/mL), indicating higher potency. The observed antibacterial activity of the germinated flaxseed extract can be attributed to the increased levels of phenolic compounds, which are known to disrupt microbial cell walls, alter membrane permeability, and interfere with enzyme function. These natural antimicrobial properties suggest potential applications in food preservation, offering a clean-label alternative to synthetic additives while enhancing the microbiological safety and shelf life of food products. Several studies have reported that flaxseed extracts exhibit strong antimicrobial activity, particularly against *S. aureus* and *E. coli*^82–84^. Gaafar et al. found that the MIC values of five flaxseed cultivars ranged from 224 to 488 µg/mL against both Gram-positive and Gram-negative bacteria^82^. The enhanced antibacterial effect is largely attributed to the high phenolic content in the extract^85^. Specifically, p-coumaric acid disrupts bacterial membranes by inhibiting the PgsA enzyme, leading to cell lysis in *S. aureus* and *E. coli*^86^. Additionally, gallic, ferulic, and cinnamic acids have been shown to exhibit bactericidal effects and inhibit *E. coli* biofilm formation and cellular activity^87^.

**Table 5:** Inhibition zone diameters and minimum inhibitory concentration (MIC) of dry flax seeds and day-5 sprouts extracts against some human-pathogenic bacteria. Gentamicin is used as a positive control for antibacterial activity.

Sample	E. coli		S. aureus	
	Inhibition zone (mm)	MIC (mg/ml)	Inhibition zone (mm)	MIC (mg/ml)
Seed	5 ± 0.1^a^	0.32 ± 0.02^a^	5 ± 0.15^a^	0.41 ± 0.01^a^
Germinated seed	13 ± 0.3^b^	0.103 ± 0.01^b^	14 ± 0.2^b^	0.103 ± 0.01^b^
Gentamicin	14 ± 0.4^b^	0.92 ± 0.03^c^	16 ± 0.3^c^	0.64 ± 0.02^c^

Values are presented as means ± SD (*n* = 4). Values with different superscript letters (a, b, c) within the same column were significant different at (*P* < 0.01).

#### Enhancement of anti-hyperglycemic properties in flaxseed through germination

Flaxseed has gained attention for its health benefits, particularly in managing type 2 diabetes. Consumption of 15 g of ground flaxseed before breakfast reduces glycemic response in diabetic men^88^, while supplementation lowers fasting blood sugar, insulin levels, and HOMA-IR, and increases QUICKI^89^. Inhibiting carbohydrate-hydrolyzing enzymes, such as α-amylase and α-glucosidase, can delay postprandial hyperglycemia^90^. This study found that the dry flaxseed and 5-day sprout extracts inhibited α-amylase and α-glucosidase in a dose-dependent manner. The 5-day sprout extract was more potent with a lower IC_50_ value for α-amylase (113.7 µg GAE/mL) and α-glucosidase (18.6 µg GAE/mL) than the dry seed extract (160 µg GAE/mL and 71 µg GAE/mL, respectively), as seen in Table 6. Flaxseed polyphenols, including sinapic acid, have been shown to inhibit these enzymes and offer anti-inflammatory and anti-glycemic effects^91^. Additionally, ferulic acid plays a key role in enzyme inhibition^92^, and combining acarbose with gallic acid enhances the inhibitory effects^93^. The 5-day flaxseed sprout extract showed notable anti-hyperglycemic activity, likely due to increased phenolic compounds that inhibit α-amylase and α-glucosidase, which help to reduce glucose absorption and support blood sugar control. This highlights the potential of germinated flaxseed as a functional ingredient for glycemic control.

**Table 6:** Antidiabetic activity of dry flax seeds and 5-day sprouts phenolic extracts.

Sample	α-amylase IC_50_ (µg GAE/ml)	α-glucosidase IC_50_ (µg GAE/ml)
Flax seeds	160 ± 4.4^a^	71 ± 2.1^a^
5-day sprouts	113.7 ± 3.1^b^	18.6 ± 0.9^b^
Acarbose	601 ± 18^c^	325 ± 11^c^

Values are presented as means ± SD (*n* = 4). Values with different superscript letters (a, b, c) within the same column were significantly different at (*P* < 0.01).

#### Industrial relevance and functional food applications

The enhancement of phenolic compounds via germination-assisted enzymatic activity offers promising applications in the food and nutraceutical industries. This process can be integrated into the large-scale production of functional ingredients by optimizing germination conditions to naturally increase bioactive content without the use of chemical additives. These phenolic-rich flaxseed products may serve as cost-effective natural antioxidants and antimicrobial agents in baked goods, beverages, and health supplements. Moreover, due to their potential antidiabetic properties, they can contribute to the development of functional foods aimed at glycemic control. This aligns with clean food trends and contributes to the production of safer, longer-lasting, and health-promoting food products.

## Conclusion

In conclusion, this study demonstrates that controlled germination of flaxseed, particularly over five days, significantly enhances its nutritional and functional properties. Using a two-level factorial design, key endogenous enzymes were optimized to promote the biosynthesis of phenolic and flavonoid compounds. These biochemical improvements were associated with increased antioxidant capacity and enriched levels of health-promoting phenolic acids. Furthermore, germinated flaxseed extracts demonstrated strong antibacterial and antidiabetic properties, supporting their potential application in the development of functional foods for enhanced health outcomes. However, the bioavailability and stability of phenolic compounds during digestion and food processing were not evaluated. Future research will be focused on the assessment of phenolic bio-accessibility and metabolism, and scale-up of the germination-enzyme process for incorporation into real food systems.

## Supplementary Information

Below is the link to the electronic supplementary material.

Supplementary Material 1

## Data Availability

The datasets generated during and/or analyzed during the current study are available from the corresponding author upon reasonable request.
