# Supplementary material for: Synergistic enzyme action boosts phenolic compounds in flaxseed during germination using a two-level factorial design
Source: Sci Rep. 2025 Nov 18;15:40384. doi: 10.1038/s41598-025-25059-4 (PMC12627697; doi:10.1038/s41598-025-25059-4)
Supplement: Supplementary file 1 — Supplementary Material 1 [file 41598_2025_25059_MOESM1_ESM.docx]

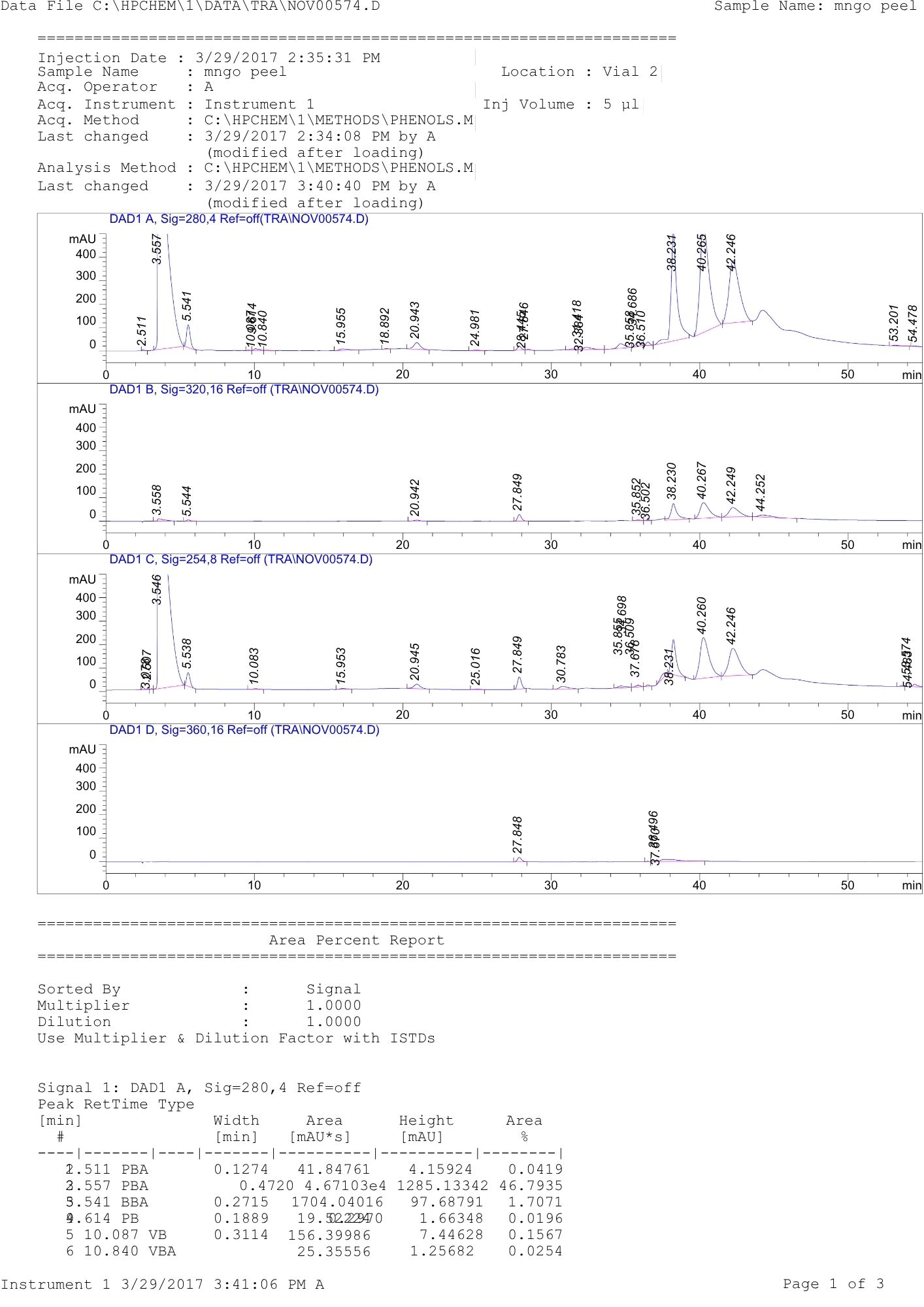


Fig. S1. HPLC chromatogram showing the phenolic profile of flaxseed extract after 5 days of germination. Peaks correspond to identified phenolic compounds as determined by retention times and standards.

### Table S1. ANOVA for the selected factorial model and Fit Statistics

| ANOVA | | | | Fit statistics for the model | | | |
| --- | --- | --- | --- | --- | --- | --- | --- |
| Responses | F-value | p-value | Model | R2 | Adjusted R2 | Predicted R2 | Adeq. precision |
| TPC | 838.94 | < 0.0001 | factorial | 0.999 | 0.9983 | 0.992 | 95.09 |
| TFC | 1124.79 | < 0.0001 | factorial | 0.999 | 0.9987 | 0.991 | 106.60 |

**
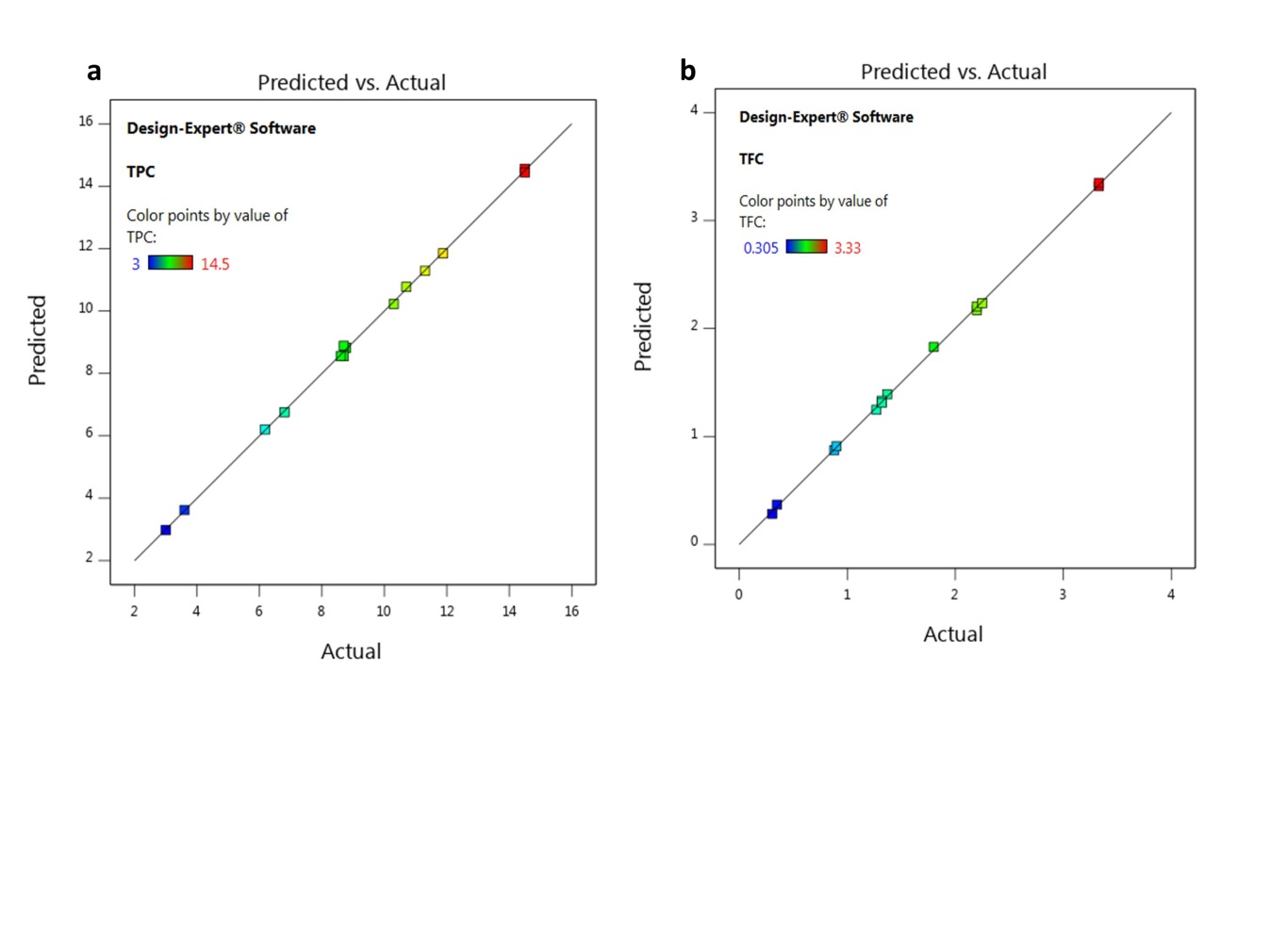
**

Fig. S2. Predicted vs. actual value graphs for (a) TPC and (b) TFC.


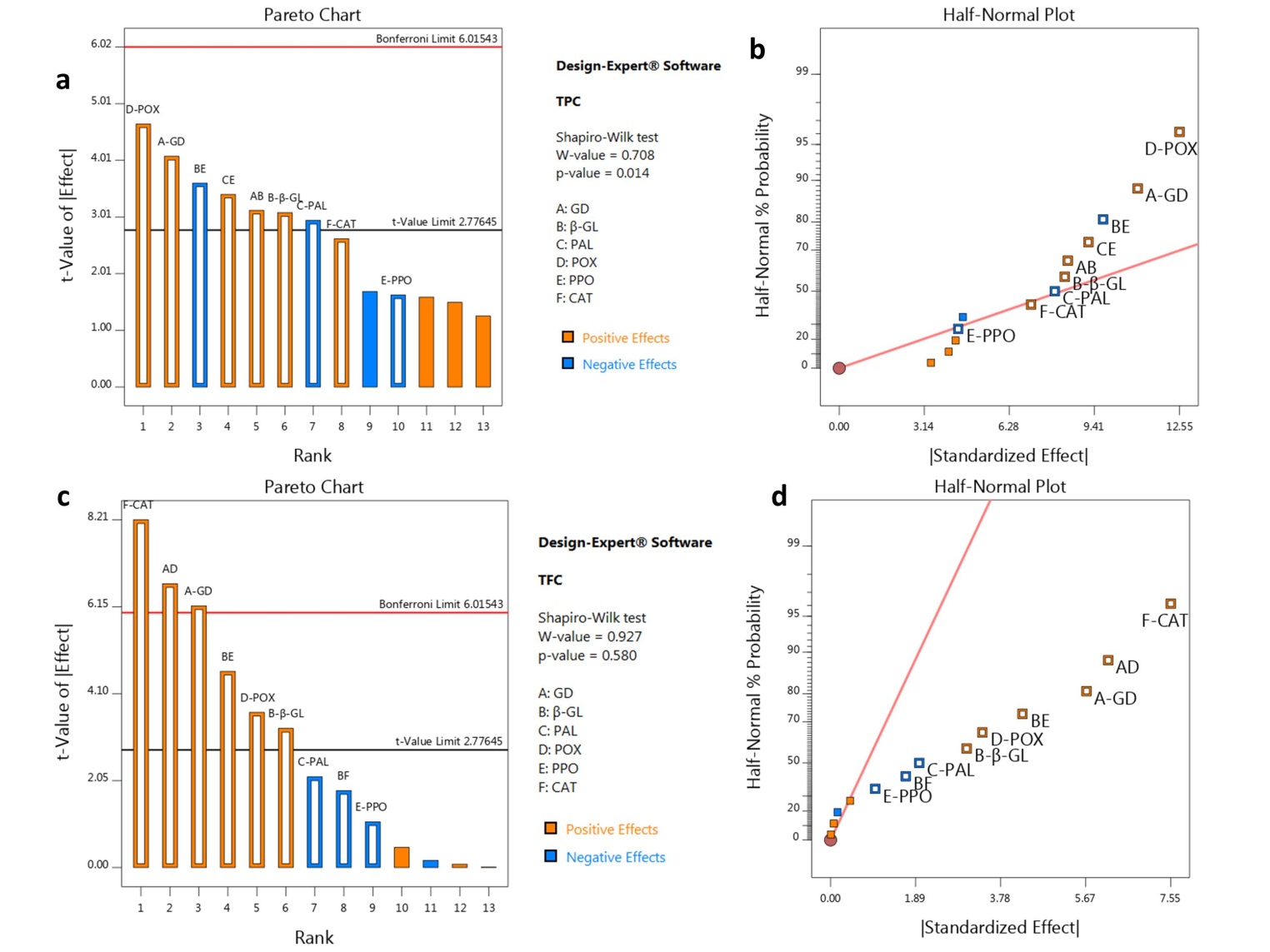


Fig. S3. Pareto charts illustrate the impact of various factors on TPC (a) and TFC (c). Factors that exert a positive influence are represented in orange, whereas those with a negative influence are depicted in blue. Additionally, half-normal charts display the effects of enzymes as variables on TPC (b) and TFC (d).

|  | **Intercept** | **GD**  **(A)** | **β-GL**  **(B)** | **PAL**  **(C)** | **POX**  **(D)** | **PPO**  **(E)** | **CAT**  **(F)** | **AB** | **AD** | **BE** | **CE** |
| --- | --- | --- | --- | --- | --- | --- | --- | --- | --- | --- | --- |
| Regression coefficients (**TPC)** | 10.180 | **5.505** | **2.354** | **-1.602** | **2.381** | -2.671 | 1.54298 | **4.409** |  | **-5.831** | **5.809** |
| **p-values**  F- **values** |  | **0.015**  **16.65** | **0.036**  **9.50** | **0.0421**  **8.68** | **0.0097**  **21.63** | 0.179  2.65 | 0.058  6.88 | **0.035**  **9.76** |  | **0.0226**  **13.01** | **0.0271**  **11.61** |
| Regression coefficients (**TFC)** | 1.068 | **2.841** | **0.929** | -0.268 | **0.579** | -0.4177 | **0.938** |  | **1.973** | **1.650** |  |
| **p-values**  F- **values** |  | **0.0035**  **38.10** | **0.0304**  **10.77** | 0.0993  4.57 | **0.0215**  **13.41** | 0.3423  1.16 | **0.0012**  **67.33** |  | **0.0026**  **44.83** | **0.0098**  **21.42** |  |

**Table S2:** ANOVA results of factorial models and regression coefficients for enzymes associated with the biosynthesis or liberation of total phenolic and flavonoid contents. Statistically significant variables are indicated in **bold** (**P** < 0.05).


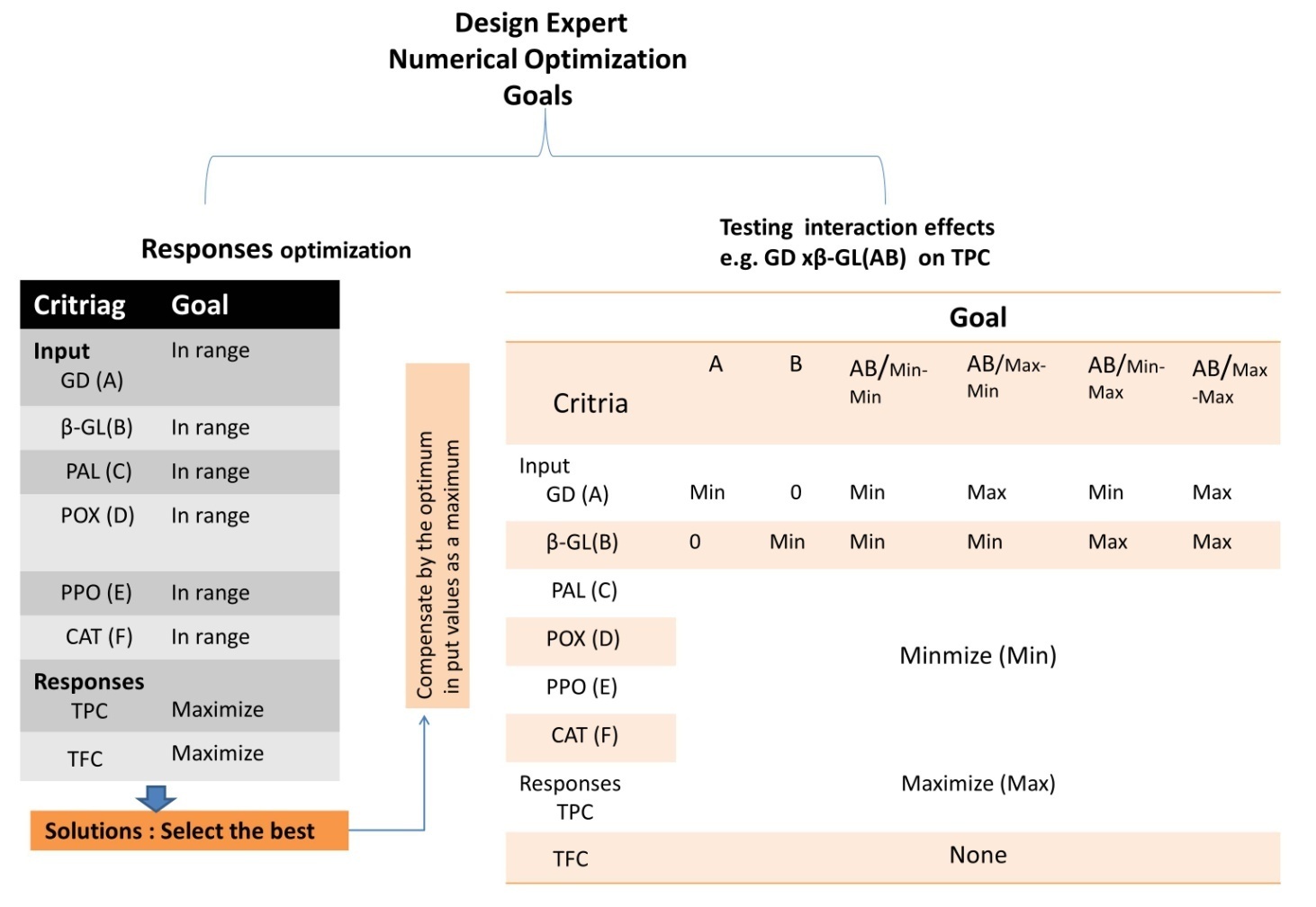


Fig S4: **Diagram for numerical optimization approaches**

#
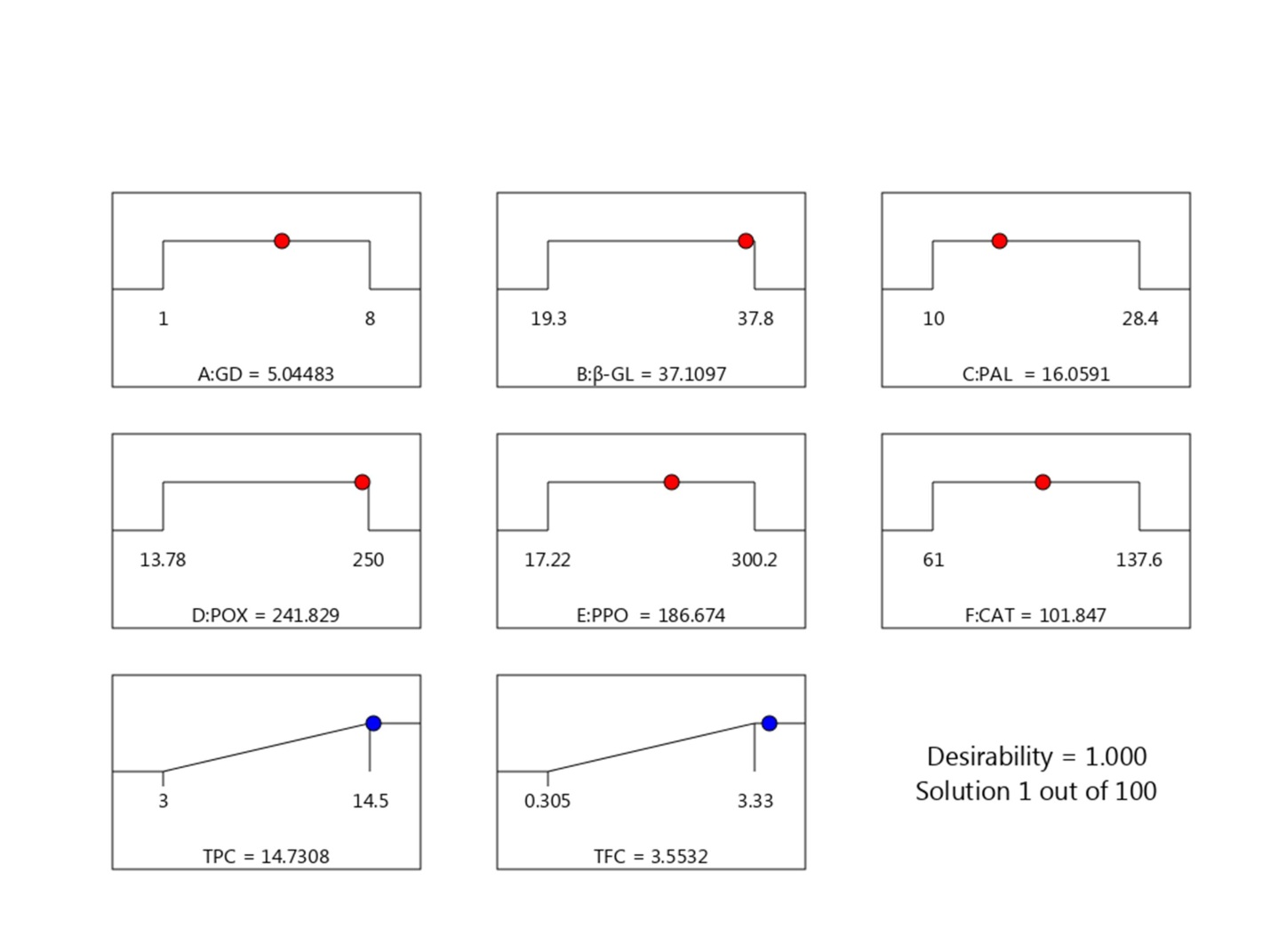


#
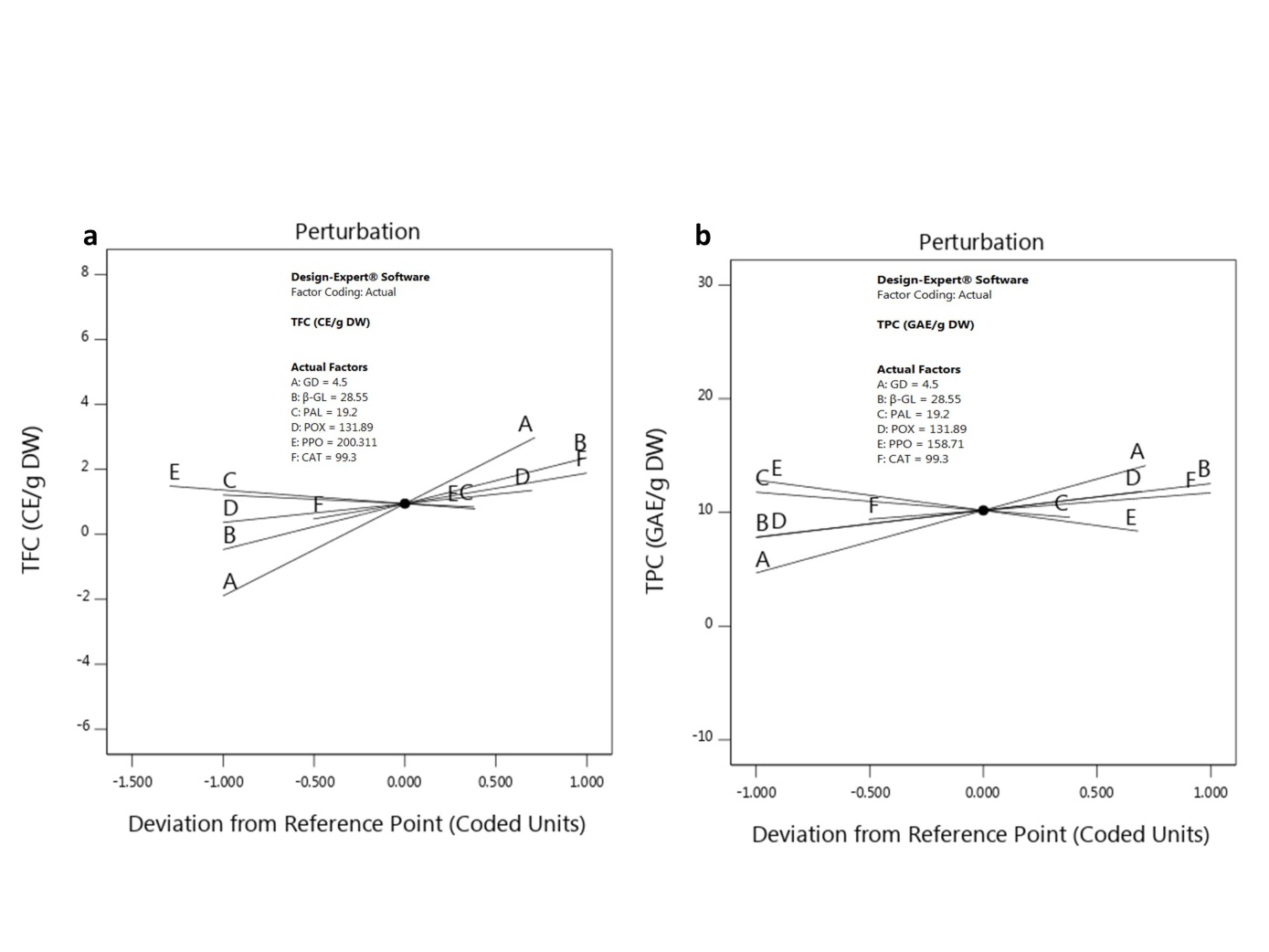


Fig.S5. Desirability ramp plots for the factors influencing the maximum production of TPC and TFC in flax sprouts.

# Table S3. presents the maximum levels of phenolic and flavonoid compounds produced by the Flax seeds during the germination process, along with the actual and predicted niche parameters associated with their production. *Relative error (%) is calculated using the formula: [(experimental value - predicted value)/experimental value] x 100.

| Measurement | Parameters | | | | | | Responses | | Desirability |
| --- | --- | --- | --- | --- | --- | --- | --- | --- | --- |
|  | GD (day) | β-GL  (U/g seed) | PAL (U/g seed) | POX  (U/g seed) | PPO  (U/g seed) | CAT  (U/g seed) | TPC (mg GAE/g | TFC  (mg CE/g) |  |
| Actual | 5 | 22.27 | 15.25 | 215.5 | 240.66 | 192.13 | 14.5 | 3.33 | 1 |
| Predicted | 5.044 | 37.1 | 16.05 | 241.09 | 186.6 | 101.84 | 14.7 | 3.55 |  |
| *Relative error% |  | | | | | | 1.38 | 6.6 |  |

|  | **TPC** | | | | | | | | | | | |
| --- | --- | --- | --- | --- | --- | --- | --- | --- | --- | --- | --- | --- |
|  | **GD xβ-GL(AB)** | | | | | | **PAL × PPO (CE)** | | | | | |
|  | Individual **GD** | Individual **β-GL** | Combination | | | | individual  **PAL** | Individual  **PPO** | Combination | | | |
| **GD (A)** | 1  Min | 0 | 1  Min | 5  Min | 1  Min | 5  Max | **Min** | | | | | |
| **β-GL (B)** | 0 | 19.3  Min | 19.3  Min | 19.3  Min | 37.1  Max | 37.1  Max |  |  |  |  |  |  |
| **PAL(C)** | **Min** | | | | | | 16.05  Min | 0 | 10  Min | 16.05  Max | 10  Min | 16.05  Max |
| **PPO (E)** |  |  |  |  |  |  | 0 | 17.22  Min | 17.22  Min | 17.22  Min | 186.6  Max | 186.6  Max |
| **Yielded TPC** | 1.64 | 1.25 | 3 | 4.92 | 5.8 | 7.2 | 3.6 | 4.2 | 7 | 5.7 | 7.9 | 9.9 |
| **desirability** | 0.84/1 | 1 | 0.94 | 0. 9 | 0.89 | 0.75 | 0.805 | 0.836 | 0.9 | 0.83 | 0.73 | 0.7 |
|  | **TFC** | | | | | | | | | | | |
|  | **GD × POX (AD)** | | | | | | **β-GL × PPO (BE)** | | | | |  |
|  | individual  **GD** | individual  **POX** | Combination | | | | individual  **β--GL** | individual  **PPO** | Combination | | |  |
| **GD (A)** | 1  Min | 0 | 1  Min | 5  Max | 1  Min | 5  Max | **Min** | | | | | |
| **POX (D)** | 0 | 13.78  Min | 13.78  Min | 13.78  Min | 241.8  Max | 241.8  Max |  |  |  |  |  |  |
| **β-GL (B)** | **Min** | | | | | | **19.3**  **Min** | **0** | 19.3  Min | 19.3  Min | 37.1 | **37.1** |
| **PPO (E)** |  |  |  |  |  |  | 0 | 17.22  Min | 17.22  Min | 186.6  Max | 17.22 | 186.6 |
| **Yielded TFC** | 0.72 | 0.86 | 1.25 | 1.9 | 1.87 | 2.7 | 0.33 | 0.9 | 1.0 | 1.76 | 2.2 | 2.73 |
| **desirability** | 0.74 | 0.8 | 0.6 | 0.74 | 0.99 | 0.9 | 0.88 | 0.85 | 0.95 | 0.82 | 0.85 | 0.91 |

**Table S4:** Interacting factors in changing concentrations varying TPC and TFC response. Min: minimum; Max: maximum concentrations


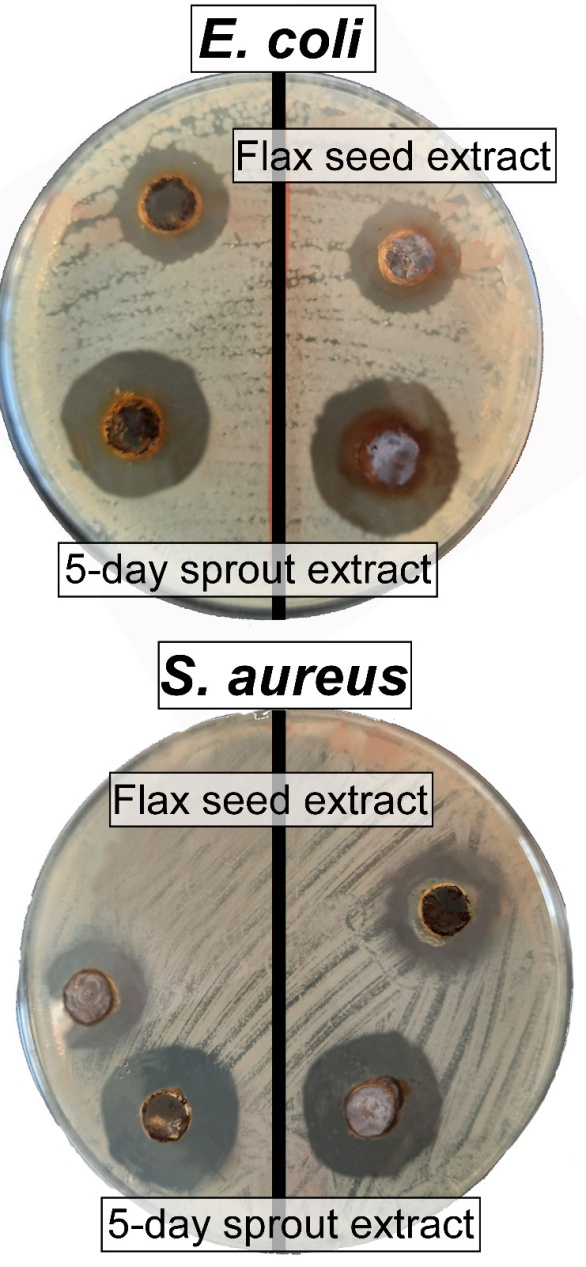


Fig S6. Inhibition zone diameters of dry flaxseed and 5-day sprout extracts against human-pathogenic *E. coli* and *S. aureus* using the agar well diffusion method.
